# Identifying and Improving Support for Caregivers of Adults with Dementia

**DOI:** 10.1002/alz70858_099340

**Published:** 2025-12-25

**Authors:** Amy Abramowitz, Jennifer Muise

**Affiliations:** ^1^ UNC, Chapel Hill, NC, USA

## Abstract

**Introduction:**

Caregiving can have a significant negative effect on the physical and mental health of caregivers, 17% of caregivers describe their own health as fair or poor compared to 10% of the general adult population. People with dementia who have caregivers experiencing a high level of stress are more likely to experience abuse, be placed in a long‐term care facility, and have behavioral and psychological symptoms of dementia (Stall et al 2019). Increasing caregiver support has been shown to reduce emergency room visits for behavioral disturbances for patients with dementia (Lau et al 2019, Gerlach et al 2023). Dementia caregivers are not systematically identified, in fact only about 20% of caregivers self‐identify as such. However over 90% of family caregivers reported that they became more proactive about seeking resources after they self‐identified (Dobrof & Ebenstein, 2003).

**Methods:**

The quality improvement project team has developed a process for identifying and screening caregivers of patients with dementia for caregiver burden within the primary care Geriatrics outpatient clinic. There are about 18 new patients per month and about 1000 patients with dementia in the Geriatrics clinic. The team generated a report in the electronic health record (EHR) to track patients with dementia in the clinic and identify new cases. Care partners were trained to screen caregivers during patient appointments using the Zarit Burden Inventory (ZBI) and all caregivers are provided with an one‐page resource guide. Caregivers who report significant burden (ZBI > 20) are then referred for care management with social work.

**Results:**

Similar to other studies, about 80% of caregivers surveyed report high caregiver burden. Caregivers, clinic staff, and clinicians are accepting and appreciative of screening outreach and resources.

**Conclusion:**

Identifying and screening caregivers in the primary care setting can connect caregivers experiencing high levels of burden with support to mitigate the risk of negative health outcomes for themselves and the patients they care for. Implementing such a process requires engaging stakeholders across the care continuum. Measuring high rates of caregiver burden can also help lead to the development of additional support resources within the health system.

